# Not all adenomyosis is equal: impact of direct adenomyosis features on live birth after ART cycles in a prospective cohort study

**DOI:** 10.3389/fendo.2026.1813673

**Published:** 2026-04-27

**Authors:** Can Benlioglu, Yavuz Emre Sukur, Bulut Varli, Erkan Kalafat, Rusen Aytac, Bülent Berker, Murat Sonmezer, Batuhan Ozmen, Cem Somer Atabekoglu

**Affiliations:** 1 Department of Obstetrics and Gynecology, Ankara University, Ankara, Türkiye; 2 Department of Obstetrics and Gynecology, Koc University, Istanbul, Türkiye

**Keywords:** adenomyosis, assisted reproduction (ART), *in vitro* fertilisation (IVF)/intracytoplasmic sperm injection, infertility, ultrasonography

## Abstract

**Objective:**

To evaluate whether the presence and type of ultrasound markers of adenomyosis, as defined by MUSA criteria, predict IVF/ICSI outcomes, specifically live birth and pregnancy loss rates.

**Methods:**

This single-center prospective cohort study included 314 women aged 18–39 years undergoing their first IVF/ICSI cycle between March 2018 and March 2020. All participants underwent standardized transvaginal ultrasound before treatment. Adenomyosis was diagnosed using MUSA criteria, distinguishing direct markers (myometrial cysts, hyperechogenic islands, echogenic subendometrial lines; *n* = 54) from indirect markers (globular enlargement, asymmetrical thickening, junctional zone irregularity; *n* = 22). Two multivariate logistic regression models were performed: (i) all adenomyosis patients versus controls (*n* = 238), and (ii) direct marker patients versus controls, adjusting for maternal age, BMI, and embryo transfer stage.

**Results:**

Among the adenomyosis group, 71.1% exhibited direct markers. Overall, adenomyosis was associated with reduced live birth per embryo transfer (28.9% vs 40.3%, aOR 0.61, 95% CI 0.34-1.08, *p* = 0.094) and significantly increased total pregnancy loss among HCG-positive patients (41.0% vs 22.6%, aOR 2.49, 95% CI 1.11-5.59, *p* = 0.026). When restricting analysis to direct markers, associations became substantially stronger: live birth rate was 22.2% (aOR 0.42, 95% CI 0.20-0.84, *p* = 0.017), representing a 58% reduction in odds, and pregnancy loss rate was 48.0% (aOR 3.77, 95% CI 1.44-10.21, *p* = 0.007), conferring a nearly 4-fold increased risk. Blastocyst-stage transfer independently improved live birth rates but did not compensate for the negative effect of direct markers.

**Conclusion:**

Direct ultrasound markers of adenomyosis identify a high-risk reproductive phenotype with substantially reduced live birth and markedly elevated total pregnancy loss risk in IVF/ICSI cycles. The presence and type of ultrasound markers, rather than adenomyosis as a binary diagnosis, should inform patient counseling and individualized treatment planning.

## 1 Introduction

Adenomyosis has been associated with various clinical entities extending from abnormal uterine bleeding to asymptomatic (1). In addition, it has been linked to subfertility in the last two decade (2, 3). As the diagnostic method shifted from histopathology to non-invasive imaging, a key clinical question has emerged: to what extent does adenomyosis, as defined by modern imaging criteria, influence reproductive outcomes in women attempting conception, particularly those undergoing assisted reproductive technologies (ART)?

Transvaginal ultrasonography (TVUS) and magnetic resonance imaging (MRI) are now the mainstays of *in vivo* diagnosis of adenomyosis. Numerous studies have either compared their diagnostic performance or explored associations between imaging-defined adenomyosis and reproductive outcomes in both spontaneous and ART pregnancies (4–16)?

To address the inter- and intra-observer variability, the Morphological Uterus Sonographic Assessment (MUSA) group proposed a standardized sonographic classification and reporting system for adenomyosis in 2015, describing features such as enlarged globular uterus, asymmetrical myometrial thickening, subendometrial echogenic lines and buds, hyperechogenic islands, myometrial cysts, and junctional zone (JZ) abnormalities including irregularity or interruption (17). Later, these markers were often called sonographic evidence of adenomyosis (SEOA) (9, 12, 17, 18). More recently, a revised MUSA consensus distinguished direct features, which are thought to reflect ectopic endometrial tissue in the myometrium (myometrial cysts, hyperechogenic islands, echogenic subendometrial lines and buds), from indirect features such as globular enlargement, asymmetrical thickening, fan-shaped shadowing and JZ irregularity (19). This conceptual shift is clinically relevant because direct features are considered more specific for adenomyosis, whereas indirect features may be less specific and overlap with other myometrial pathologies like aging uterus.

Prospective data using these revised definitions in women undergoing ART are now emerging. In a large 3D TVUS study of women scheduled for their first IVF/ICSI treatment, at least one direct or indirect MUSA feature of adenomyosis was identified in approximately one quarter of women (23.4%), while direct features were present in about 10% (9.6%) (20). In a subsequent prospective cohort from the same group, the presence of direct features, as defined by the revised MUSA consensus, was associated with a significantly lower cumulative live birth rate after the first IVF/ICSI treatment and a higher risk of miscarriage after frozen embryo transfer (FET) (21). These data suggest that direct features capture a subgroup of women with clinically meaningful impairment in IVF prognosis.

The present prospective cohort study aimed to assess whether the presence of MUSA adenomyosis features on pre-treatment transvaginal ultrasound is associated with subsequent IVF/ICSI outcomes, specifically total pregnancy loss and live birth rates in women undergoing ART.

## 2 Materials and methods

### 2.1 Study design and ethical approval

The study was a single-center prospective cohort study conducted at a university-based infertility clinic between March 2018 and March 2020. The Ethical Committee of Institutional Clinical Trial Unit approved the study (12.03.2018; 05-311-18). Written informed consent was obtained from all included patients. 18–39 years old patients admitted to the infertility out-patient clinic of Ankara University School of Medicine and planned to start an IVF/ICSI cycle were included. All couples underwent a standardized infertility work-up including medical history, physical examination, hormonal assessment, semen analysis, transvaginal sonography (TVS), and hysterosalpingography (HSG). Women with untreated hydrosalpinx or clinically significant intracavitary pathology, including FIGO type 0, 1, or 2 submucosal myomas, were not included in the study cohort unless these lesions had been surgically managed before ART. Couples with severe male factor infertility, such as azoospermia and patients with sonographically diagnosed with rASRM Grade 3–4 endometriosis were excluded. All women included in the study had primary infertility.

### 2.2 Sonographic evaluation

The TVS examination was conducted using a 7.5 MHz ultrasound probe, and sonographic assessments were standardized. First, the transverse plane of the uterus was examined and was followed by an evaluation of the cervical canal and uterine cavity. Afterward, the uterus and endometrium were assessed on the longitudinal axis by rotating the probe 90 degrees counterclockwise. Myometrium was systematically evaluated for the presence of any abnormality. It was recorded in three-dimensional coordinates if there was any uterine fibroid or other pathology. Subsequently, tubal and adnexal evaluations were made by starting with the interstitial parts of the tube on the coronal axis. While observing, cases with signs of hydrosalpinx, endometrioma, or any other pelvic pathology were dimensioned and carefully noted.

Myometrial asymmetry, myometrial heterogeneity, myometrial cysts, loss of junctional zone (JZ), linear striations, globular enlargement of the uterus, and endometrial islands (endometrial lakes-hyperechogenic islands) were selected as sonographic features. These definitions were determined based on MUSA criteria (17, 19). The presence of any marker was noted as direct and indirect features.

All transvaginal sonographic evaluations were performed by two experienced clinicians (Y.E.S., B.V.), each with at least 10 years of experience in gynecologic ultrasound assessment. In cases of disagreement regarding adenomyosis features, scans were reevaluated by a third senior clinician (C.S.A.). As all ultrasound examinations were conducted just before oocyte-pick up (OPU), the operators were not aware of subsequent reproductive outcomes at the time of assessment.

### 2.3 IVF cycles and outcome parameters

IVF/ICSI cycles were undertaken using formerly represented protocols (22). Briefly, GnRH antagonist down-regulation protocol and single frozen embryo transfer with luteal phase support (with vaginal micronized progesterone) was commenced in all patients in the present study. All embryos were morphologically assessed by the same senior embryologists using the Gardner blastocyst grading system (23). Good-quality blastocysts were defined as those reaching at least expansion grade 3 with an inner cell mass and trophectoderm score of A or B (24). No embryos in this cohort underwent preimplantation genetic testing for aneuploidy (PGT-A). Only the first embryo transfer following the index IVF/ICSI cycle was included in the analysis. Clinical pregnancy was defined as the presence of a fetal heartbeat on transvaginal ultrasound at approximately 6 weeks of gestation. Total pregnancy loss was defined as any spontaneous loss occurring after a positive biochemical pregnancy test. Live birth was defined as the delivery of a live infant at or beyond 24 weeks of gestation.

### 2.4 Statistical analysis and outcome measures

Data analysis was performed using R software (version 4.5.2) with the relevant packages like ggplot2, finalfit. Baseline characteristics were compared between control and adenomyosis groups using the Mann-Whitney U test for continuous variables and chi-square test (or Fisher’s exact test when appropriate) for categorical variables. Assuming live birth rates of 25% and 40% in women with and without adenomyosis, respectively, a minimum total sample size of 302 women was required to achieve 80% power with a two-sided alpha level of 0.05.

To evaluate the independent effect of adenomyosis on reproductive outcomes, two separate multivariate logistic regression analyses were performed: All adenomyosis patients versus control and direct marker adenomyosis patients versus control (excluding indirect marker cases). Both models were adjusted for clinically relevant covariates: age, BMI, and embryo transfer stage (cleavage vs. blastocyst). Variables were selected based on clinical relevance and univariate associations with the outcome. Primary outcomes were live birth rate per embryo transfer and total pregnancy loss rate among biochemical pregnancy-positive patients. Results are reported as adjusted odds ratios (aOR) with 95% confidence intervals. Statistical significance was set at p < 0.05 for all analyses.

## 3 Results

### 3.1 Baseline characteristics

A total of 314 patients were included in the final analysis: 238 controls and 76 patients (24.2%) with adenomyosis (study flowchart - **Supplementary Figure 1**). Among the adenomyosis group, 54 patients (71.1%) exhibited direct ultrasound markers and 22 (28.9%) had only indirect markers. Baseline patient and cycle characteristics are summarized in **Table 1**. The adenomyosis group was slightly older (34.4 ± 2.9 vs 33.8 ± 1.6 years, p=0.021) and had lower BMI (25.5 ± 1.2 vs 26.1 ± 2.4 kg/m², p=0.029) than controls. No significant differences were observed in ovarian reserve markers (AMH: 1.1 ± 0.4 vs 1.0 ± 0.2 ng/mL, p=0.697; basal FSH: 6.8 ± 0.6 vs 6.8 ± 0.3 IU/L, p=0.899), total gonadotropin dose, peak estradiol levels, or embryological parameters including maturation rate (82.2 ± 12.6% vs 83.8 ± 10.3%, p=0.269) and fertilization rate (83.4 ± 14.8% vs 80.8 ± 15.0%, p=0.189). The proportion of blastocyst-stage transfers was similar between groups (78.9% vs 76.5%, p=0.771).

**Table 1:** The comparison of baseline demographic and cycle characteristics between adenomyosis and control groups.

	Control	Adenomyosis	p
Age (years)	33.8 (1.6)	34.4 (2.9)	**0.021**
BMI (kg/m²)	26.1 (2.4)	25.5 (1.2)	**0.029**
AMH (ng/mL)	1.0 (0.2)	1.1 (0.4)	0.697
Basal FSH (IU/L)	6.8 (0.3)	6.8 (0.6)	0.899
Infertility Duration (years)	2.8 (0.6)	2.8 (0.6)	0.411
Total Gonadotropin Dose (IU)	1981.0 (389.8)	2028.9 (492.0)	0.383
E2 on Trigger Day (pg/mL)	2414.5 (187.9)	2412.7 (270.0)	0.951
COC count (total)	10.0 (2.0)	9.6 (2.2)	0.165
MII count (total)	8.3 (2.0)	7.9 (2.1)	0.068
Maturation Rate (%)	83.8 (10.3)	82.2 (12.6)	0.269
2PN count (total)	6.7 (2.0)	6.6 (2.2)	0.524
Fertilization Rate (%)	80.8 (15.0)	83.4 (14.8)	0.189
Embryo Transfer			
Day 3	56 (23.5)	16 (21.1)	0.771
Day 5	182 (76.5)	60 (78.9)	
Endometrial Thickness (mm)	9.3 (0.6)	9.4 (0.7)	0.405

BMI, Body Mass Index; AMH, anti-Mullerian Hormone; FSH, Follicle Stimulating Hormone; COC, cumulus oophorus complex; MII, Metaphase II; PN, pronuclei.

Bold values show statistically significant (p < 0..05).

### 3.2 Clinical outcomes

**Figure 1:**
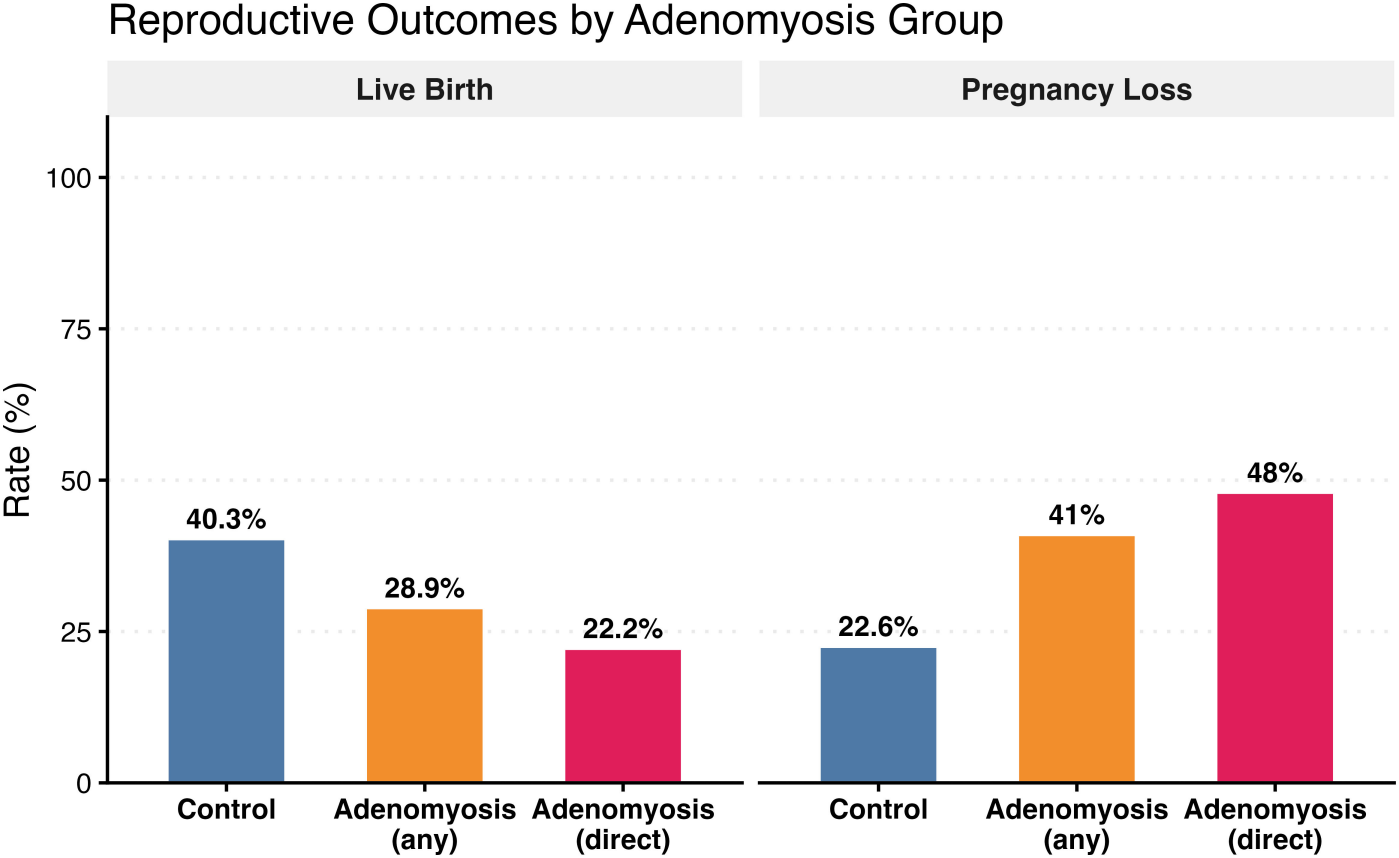
Live birth and total pregnancy loss rates by adenomyosis group. Bar chart showing crude live birth rates (left panel) and total pregnancy loss rates among biochemical pregnancy-positive patients (right panel) across three groups: controls (*n* = 238), all adenomyosis patients regardless of marker type (*n* = 76), and adenomyosis patients with direct ultrasound markers only (*n* = 54). Percentages are displayed above each bar. Total pregnancy loss rates were calculated among the 172 patients who achieved biochemical pregnancy (HCG-positive).

**Figure 2:**
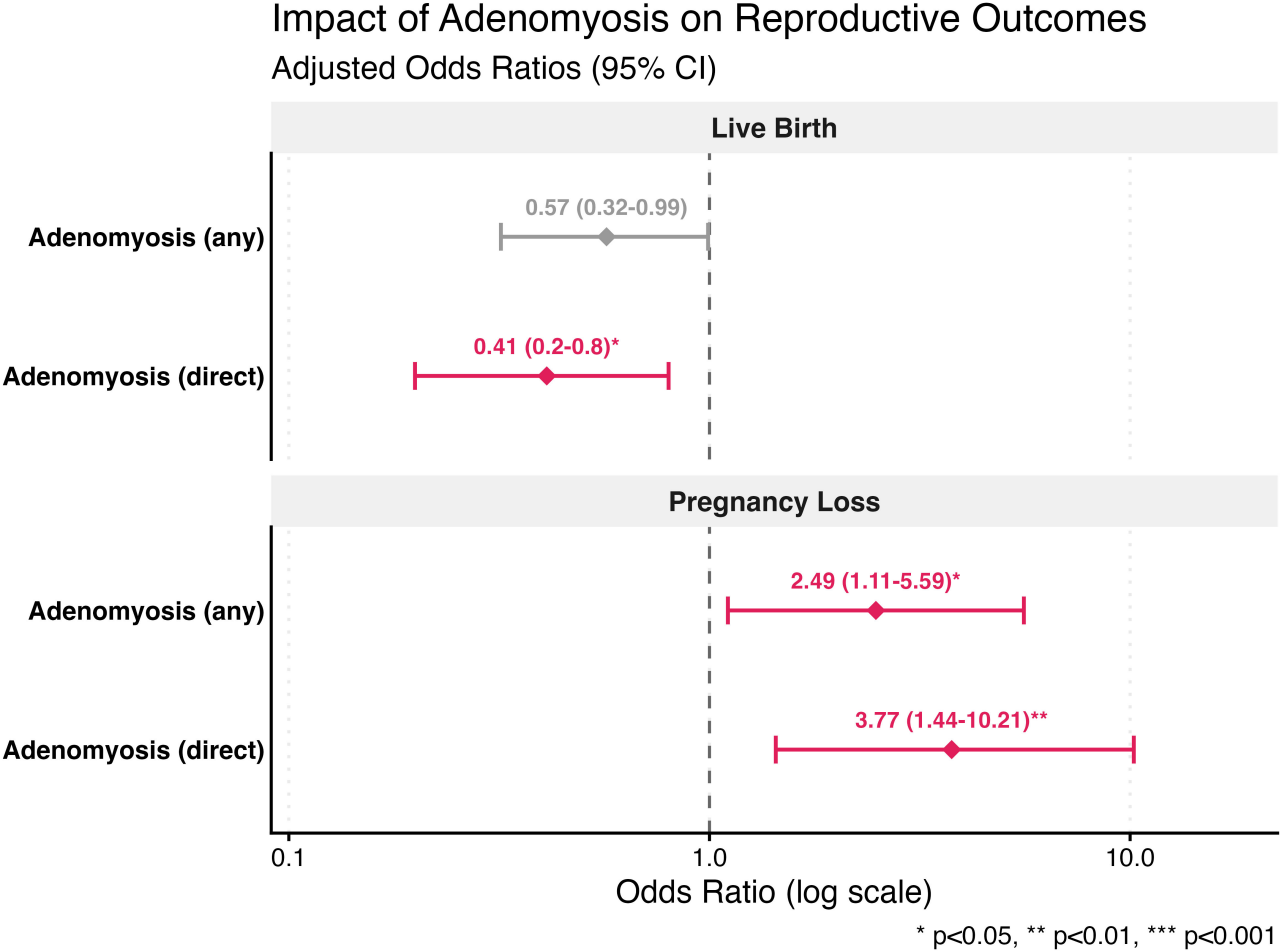
Impact of adenomyosis on live birth and total pregnancy loss: adjusted odds ratios stratified by marker type. Forest plot displaying adjusted odds ratios (aOR) with 95% confidence intervals for live birth and total pregnancy loss outcomes. Two comparisons are shown: all adenomyosis patients (combining direct and indirect ultrasound markers) versus controls, and direct marker adenomyosis patients only versus controls. Models were adjusted for maternal age, BMI, and embryo transfer stage (cleavage vs. blastocyst). The vertical dashed line represents the null effect (OR = 1.0). Red diamonds indicate statistically significant associations (*p* < 0.05); gray diamonds indicate non-significant associations. *p < 0.05; **p < 0.01.

The overall live birth rate was lower in the adenomyosis group compared to controls (28.9% vs 40.3%), though this did not reach statistical significance in univariate analysis (OR 0.60, 95% CI 0.34-1.04, p=0.076) (**Figure 1**). After adjustment for age, BMI, and embryo transfer stage, adenomyosis remained associated with reduced live birth, though the effect was marginally non-significant (aOR 0.61, 95% CI 0.34-1.08, p=0.094) (**Table 2**, **Figure 2**). When restricting the analysis to patients with direct ultrasound markers of adenomyosis versus controls, the negative impact on live birth became more pronounced and statistically significant. The live birth rate was 22.2% in the direct marker group compared to 40.3% in controls (OR 0.42, 95% CI 0.20-0.82, p=0.015) (**Figure 1**). This association persisted after multivariable adjustment (aOR 0.42, 95% CI 0.20-0.84, p=0.017), representing a 58% reduction in the odds of live birth (**Table 2**, **Figure 2**). Blastocyst-stage transfer was independently associated with improved live birth outcomes in both analyses (all adenomyosis: aOR 2.14, 95% CI 1.17-4.04, p=0.016; direct marker analysis: aOR 2.41, 95% CI 1.27-4.76, p=0.009). Increasing maternal age showed a trend toward reduced live birth in multivariable models (all adenomyosis: aOR 0.88 per year, 95% CI 0.77-0.99, p=0.042; direct marker analysis: aOR 0.86 per year, 95% CI 0.75-0.99, p=0.037).

**Table 2:** Univariable and multivariable logistic regression for live birth in women with any adenomyosis and with direct MUSA features versus controls.

Dependent: Live birth	0	1	OR (univariable)	OR (multivariable)
All adenomyosis vs Control				
Age (years)	34.1 (2.1)	33.7 (1.7)	0.92 (0.82-1.03, p=0.146)	**0.88 (0.77-0.99, p=0.042)**
BMI (kg/m²)	26.0 (2.3)	25.9 (2.0)	0.96 (0.86-1.07, p=0.472)	0.95 (0.85-1.06, p=0.367)
Embryo Transfer (day 3 vs day 5)	52 (72.2)	20 (27.8)	-	-
	144 (59.5)	98 (40.5)	1.77 (1.01-3.21, p=0.052)	**2.14 (1.17-4.04, p=0.016)**
Adenomyosis	142 (59.7)	96 (40.3)	-	-
	54 (71.1)	22 (28.9)	0.60 (0.34-1.04, p=0.076)	0.61 (0.34-1.08, p=0.094)
Direct Markers of Adenomyosis vs Control				
Age	34.1 (2.1)	33.7 (1.7)	0.90 (0.80-1.02, p=0.111)	**0.86 (0.75-0.99, p=0.037)**
BMI	26.1 (2.3)	25.9 (2.0)	0.96 (0.86-1.07, p=0.471)	0.95 (0.85-1.06, p=0.362)
Embryo Transfer (day 3 vs day 5)	48 (73.8)	17 (26.2)	-	-
	136 (59.9)	91 (40.1)	**1.89 (1.04-3.57, p=0.042)**	**2.41 (1.27-4.76, p=0.009)**
Direct Marker	142 (59.7)	96 (40.3)	-	-
	42 (77.8)	12 (22.2)	**0.42 (0.20-0.82, p=0.015)**	**0.42 (0.20-0.84, p=0.017)**

Bold values show statistically significant (p < 0..05).

Among the 172 patients who had positive biochemical pregnancy, the total pregnancy loss rate was significantly higher in the adenomyosis group (41.0% vs 22.6%, OR 2.39, 95% CI 1.11-5.09, p=0.024) (**Figure 1**). After adjustment for age, BMI, and embryo transfer stage, adenomyosis remained a significant predictor of pregnancy loss (aOR 2.49, 95% CI 1.11-5.59, p=0.026), representing a 2.5-fold increased risk (**Table 3**, **Figure 2**). The association was even stronger when examining only patients with direct ultrasound markers. In this subgroup, the pregnancy loss rate reached 48.0% compared to 22.6% in controls (OR 3.17, 95% CI 1.30-7.72, p=0.011). In the adjusted model, direct markers conferred a nearly 4-fold increased risk of pregnancy loss (aOR 3.77, 95% CI 1.44-10.21, p=0.007) (**Table 3**, **Figure 2**). Neither maternal age nor BMI demonstrated significant associations with pregnancy loss risk in any of the models. Blastocyst-stage transfer showed a trend toward lower pregnancy loss rates, though this did not reach statistical significance (all adenomyosis: aOR 0.50, 95% CI 0.21-1.20, p=0.116; direct marker analysis: aOR 0.49, 95% CI 0.19-1.29, p=0.142).

**Table 3:** Univariable and multivariable logistic regression for total pregnancy loss in women with any adenomyosis and with direct MUSA features versus controls.

Dependent: Total pregnancy loss	0	1	OR (univariable)	OR (multivariable)
All adenomyosis vs Control				
Age (years)	33.7 (1.7)	33.6 (2.2)	0.98 (0.81-1.18, p=0.791)	0.98 (0.80-1.20, p=0.850)
BMI (kg/m²)	25.8 (2.0)	25.9 (2.4)	1.03 (0.87-1.20, p=0.748)	1.04 (0.88-1.23, p=0.623)
Embryo Transfer (day 3 vs day 5)	22 (61.1)	14 (38.9)	-	-
	104 (76.5)	32 (23.5)	0.48 (0.22-1.07, p=0.067)	0.50 (0.21-1.20, p=0.116)
Adenomyosis	103 (77.4)	30 (22.6)	-	-
	23 (59.0)	16 (41.0)	**2.39 (1.11-5.09, p=0.024)**	**2.49 (1.11-5.59, p=0.026)**
Direct Markers of Adenomyosis vs Control				
Age	33.7 (1.6)	33.6 (2.1)	0.96 (0.79-1.18, p=0.711)	0.95 (0.75-1.19, p=0.634)
BMI	25.8 (2.0)	26.0 (2.4)	1.04 (0.88-1.22, p=0.664)	1.06 (0.89-1.26, p=0.527)
Embryo Transfer (day 3 vs day 5)	19 (61.3)	12 (38.7)	-	-
	97 (76.4)	30 (23.6)	0.49 (0.21-1.14, p=0.092)	0.49 (0.19-1.29, p=0.142)
Direct Marker	103 (77.4)	30 (22.6)	-	-
	13 (52.0)	12 (48.0)	**3.17 (1.30-7.72, p=0.011)**	**3.77 (1.44-10.21, p=0.007)**

Bold values show statistically significant (p < 0..05).

## 4 Discussion

The present prospective cohort study evaluated the impact of MUSA-defined adenomyosis features on IVF/ICSI outcomes and demonstrates that not all sonographic patterns of adenomyosis carry the same reproductive risk. In the overall adenomyosis group, we observed a lower live birth rate compared with controls, although this association remained of borderline statistical significance after adjustment for age, BMI and embryo transfer stage, whereas pregnancy loss rates were clearly higher in women with adenomyosis. When analyses were restricted to patients exhibiting direct ultrasound markers, the adverse associations became more pronounced; direct features were associated with an approximately 60% reduction in the odds of live birth and a nearly four-fold increase in pregnancy loss risk compared with women without adenomyosis, even after multivariable adjustment. In contrast, the inclusion of women with only indirect markers appeared to attenuate these effects, suggesting that this subgroup may dilute the overall impact of adenomyosis on IVF/ICSI prognosis. Blastocyst-stage transfer independently improved live birth rates across models but did not fully compensate for the negative effect of direct adenomyosis markers. Taken together, these findings indicate that direct MUSA features identify a higher risk adenomyosis phenotype with clinically meaningful implications for counselling and treatment planning in IVF/ICSI cycles. However, we also acknowledge that that sonographic phenotypes characterized by direct MUSA features may identify a subgroup with poorer reproductive prognosis, rather than proving a distinct causal or histologically confirmed disease subtype.

The detrimental effects of adenomyosis were demonstrated in five meta-analyses and systematic reviews (13, 25–28); however, conflicting results also exist (12, 29). Notably, most of the studies in the current literature have assessed ART outcomes based on the presence or absence of any sonographic feature of adenomyosis rather than disentangling the prognostic weight of individual markers or distinguishing between direct and indirect MUSA features. In a Mavrelos et al.’s study, they concluded that adenomyosis had a cumulative effect on clinical outcomes, and the addition of each sonographic sign decreased clinical pregnancy rates (9). On the other hand, Higgins et al. underlined that even though three MUSA criteria were used to diagnose adenomyosis, there was no significant correlation between adenomyosis and live birth rates (12).

Within the IVF literature, our findings sit between the reassuring and the more pessimistic signals emerging from recent large cohorts and donor-cycle data. In the HRT-FET setting, the retrospective study by Sachs-Guedj et al. reported that women with adenomyosis defined according to MUSA had significantly lower clinical pregnancy and live birth rates and higher miscarriage rates compared with controls, even after extensive adjustment for age, use of donor oocytes, PGT-A, freeze-all strategy and pre-treatment with GnRH agonists (aOR for live birth 0.46; aOR for miscarriage 2.13 (15). Donor-oocyte models, by contrast, largely remove embryo aneuploidy, ovarian reserve and age as confounders and therefore provide a purer readout of the uterine contribution. In this context, the prospective single-embryo-transfer donor study by Cozzolino et al. showed that, although overall implantation, clinical pregnancy and live birth rates were comparable between recipients with and without adenomyosis, miscarriage rates were significantly higher when adenomyosis was present, with the strongest excess risk in women with direct junctional zone involvement, diffuse and severe disease; intriguingly, features confined to the outer myometrium were associated with better ongoing pregnancy rates. Feferkorn & Tulandi commented on the Dason et al.’s donor cycle study findings in their editorial, emphasizing the importance of considering both direct and indirect features of adenomyosis when using MUSA criteria and highlighting the potential for overdiagnosis, which could result in unnecessary treatments and patient distress (14, 30). Our data broadly confirm this pattern of reduced live birth and increased miscarriage, but refine it by showing that the excess risk is largely concentrated in women with direct sonographic markers: when adenomyosis is defined simply as “any MUSA feature”, the effect on live birth is modest and of borderline significance, whereas focusing on direct markers alone reveals a nearly 60% reduction in the odds of live birth and an almost four-fold increase in miscarriages. Taken together, the donor-cycle literature suggests that adenomyosis may exert its main impact by creating a subset of high-risk uterine phenotypes (those captured by direct MUSA features and by junctional zone involvement) that are particularly prone to early pregnancy loss. Our results, showing a relatively modest effect of “any MUSA feature” but a marked reduction in live birth and sharp rise in miscarriage when direct signs are present, align with this emerging view. At the same time, we acknowledge the reassuring cumulative data from a recent large prospective cohort in which women with endometriosis and/or adenomyosis, diagnosed using the IDEA and revised MUSA definitions, experienced a 20-25% relative reduction in cumulative live birth over three IVF/ICSI cycles but still achieved CLBRs in intention to treat analysis which was 53.2% compared with 70% in unaffected women (31). In light of these findings, even though direct MUSA features identify a subgroup with poorer per-cycle prognosis and increased miscarriage risk, repeated high-quality IVF/ICSI treatment still offers a substantial chance of live birth and should not be withheld solely because adenomyosis is diagnosed on ultrasound.

The major strength of this study was its prospective design. In addition, the systematic exploration of individual parameters and the multivariate approach may add credence to our observations. Furthermore, all pregnant patients were followed up until the end of the pregnancy to evaluate the possible pathophysiologic effects of adenomyosis on live birth chance. The present study shows that careful evaluation of adenomyosis on sonographic scans may improve the counseling of infertile women considering IVF/ICSI–ET. The major limitation of the present study was the limited sample size which can result in type-2 errors. Other important limitations of the present study include the possibility of residual confounding and the use of two-dimensional transvaginal sonography (2D TVS) for adenomyosis evaluation. Although several clinically and biologically plausible confounders were addressed through methodological restrictions and statistical adjustment, residual confounding may still have influenced the observed outcomes. Accordingly, while the observed associations were clinically meaningful, the observational design of the study precludes causal inference. An additional limitation is that the subgroup with indirect adenomyosis features only was relatively small. Therefore, no further subgroup analyses were performed for this group in order to avoid overinterpretation and speculation based on imprecise estimates. Further adequately powered studies are needed to specifically evaluate and compare reproductive outcomes between direct and indirect adenomyosis phenotypes.

## 5 Conclusion

This prospective cohort study suggests that direct ultrasound markers of adenomyosis, as defined by MUSA criteria, identify a high-risk reproductive phenotype characterized by substantially reduced live birth rates and markedly elevated pregnancy loss risk in IVF/ICSI cycles. Women showing direct sonographic features experienced an approximately 60% reduction in the odds of live birth and a nearly four-fold increase in total pregnancy loss compared with controls, even after adjustment for age, BMI, and embryo transfer stage. In contrast, when adenomyosis was defined broadly to include indirect markers, the associations were attenuated, indicating heterogeneity in reproductive risk across sonographic adenomyosis phenotypes. These findings suggest that ultrasound phenotype, rather than adenomyosis as a simple binary diagnosis, may better inform patient counseling and individualized treatment planning.

## Data Availability

The original contributions presented in the study are included in the article/**Supplementary Material**. Further inquiries can be directed to the corresponding author.
