# Supplementary figures and images for: Not all adenomyosis is equal: impact of direct adenomyosis features on live birth after ART cycles in a prospective cohort study
Source: Front Endocrinol (Lausanne). 2026 Apr 27;17:1813673. doi: 10.3389/fendo.2026.1813673 (PMC13158101; doi:10.3389/fendo.2026.1813673)

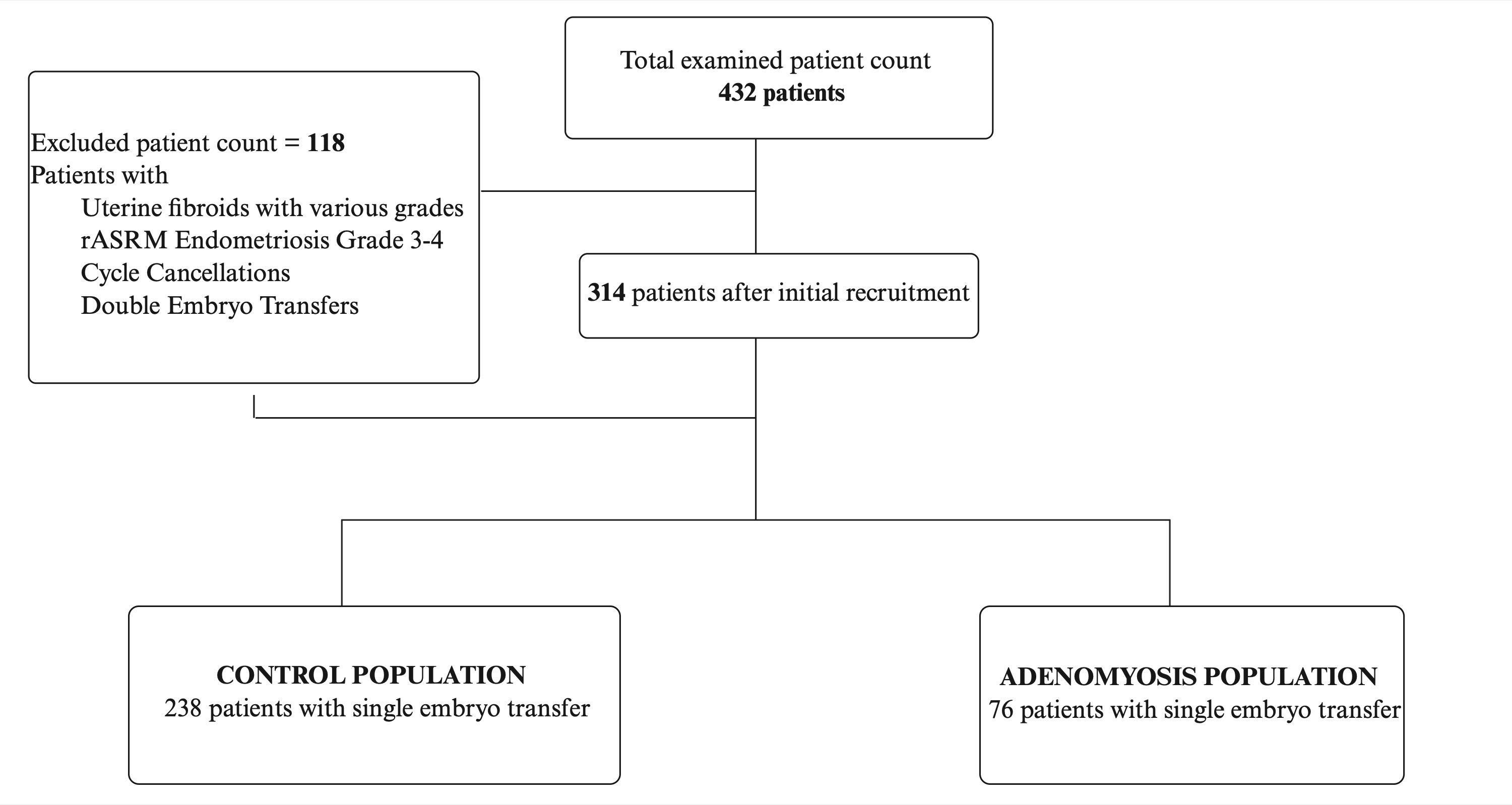

Supplement: Supplementary file 1 [file Image1.jpeg]
